# Exercise Testing and Prescription in Patients with Congenital Heart Disease

**DOI:** 10.1155/2010/791980

**Published:** 2010-09-06

**Authors:** A. D. J. ten Harkel, T. Takken

**Affiliations:** ^1^Department of Pediatric Cardiology, Leiden University Medical Center, Albinusdreef 2, P.O. Box 9600, 2300 RC Leiden, The Netherlands; ^2^Child Development and Exercise Center, Wilhelmina Children's Hospital, UMC Utrecht, 3508 AB Utrecht, The Netherlands

## Abstract

The present paper provides a review of the literature regarding exercise testing, exercise capacity, and the role of exercise training in patients with congenital heart disease (CHD). Different measures of exercise capacity are discussed, including both simple and more advanced exercise parameters. Different groups of patients, including shunt lesions, pulmonary valvar stenosis, patients after completion of Fontan circulation, and patients with pulmonary arterial hypertension are discussed separately in more detail. It has been underscored that an active lifestyle, taking exercise limitations and potential risks of exercise into account is of utmost importance. Increased exercise capacity in these patients is furthermore correlated with an improvement of objective and subjective quality of life.

## 1. Introduction

In the present era most patients (85%) with congenital heart disease (CHD) reach adulthood [1]. It has therefore become of utmost importance to maintain a good quality of life and physical fitness for these patients. Many studies have established a moderate to good correlation between quality of life and exercise capacity [2, 3]. There are, however, many factors that impede exercise capacity in patients with CHD. They usually perform less physical activities as compared to normal controls [4], know little about their own possibilities [5], and largely overestimate their exercise capacity [6]. In addition, overprotection from the environment results in further reductions in physical activity [7].

We therefore reviewed the literature regarding the assessment of exercise capacity, using both simple and more advanced methods in patients with CHD, the exercise capacity of patients with CHD, and possibilities of exercising without considerable risks.

## 2. Exercise Testing

Exercise testing is a valuable tool in the management of pediatric patients with heart disease. It can be used to help determine the need for medical or surgical interventions and can be used to determine the efficacy of these interventions [8–10]. Measurement of exercise capacity and other physiological responses provides objective information about the functional status of heart, lungs, and peripheral muscles. This information can be of value in making clinical decisions resulting in a reduced use of hospital facilities, and improved functional capacity and quality of life. A list of indications for exercise testing is provided in Table 1.

 Although emergencies are rare in pediatric exercise testing [9], staff should be familiar with emergency maneuvers and the criteria when an exercise test should be terminated (Table 2). Technical aspects of exercise testing are beyond the scope of this paper and can be found in the comprehensive article by Paridon et al. [12].

## 3. Cardiopulmonary Responses to Exercise

During graded exercise testing, many variables can be measured. The simplest measures are the assessment of heart rate (HR) and work rate or endurance time. The measurement of HR during exercise is simple and inexpensive and provides important information about the cardiovascular system. During exercise, HR increases and the highest obtained HR is defined as the HR_peak_. In healthy children HR_peak_ is typically about 200 ± 10 for treadmill testing and 195 ± 10 for cycle ergometer testing [9]. After cessation of exercise HR returns to the baseline. This HR recovery is believed to be mainly influenced by vagal autonomic activity, Body Mass Index, and aerobic capacity [14]. Most studies demonstrate a more rapid decline in HR after cessation of exercise in younger children [14, 15]. Studies that determined stroke volume (SV) show that children have a smaller SV during maximal exercise compared to adults. Up to moderate submaximal exercise SV increases, but further increase in cardiac output (CO) during increasing exercise intensity is in children regulated by an increase in HR [9]. This means that beyond moderate intensity exercise (approximately 40% of VO_2peak_) a reduced increase in HR will result in reduced CO and, although partially compensated by a larger arteriovenous oxygen difference, a reduction in peak oxygen uptake [16].

 When a respiratory gas analysis system is available, ventilation (VE), oxygen uptake (VO_2_), and carbon dioxide production (VCO_2_) can be measured continuously. Peak oxygen uptake (VO_2peak_) is the traditional gold-standard of aerobic capacity and is a widely used parameter. This VO_2peak_ is determined by the maximal rate of the oxygen transport from lungs to muscle [16], and may be limited by SV, HR, or tissue extraction. Normal values for VO_2peak_ for children and adolescents are recently published by ten Harkel et al. [17].

Ventilatory anaerobic threshold (VAT) is another important parameter of exercise capacity. It is defined as the point at which minute ventilation increases disproportionally relative to VO_2_, usually occurring at 50–70% of VO_2peak_ [17]. VAT reflects the point at which anaerobic metabolism starts to increase since oxygen supply cannot keep up with the increasing metabolic requirements of exercising muscles [16].

 There are several exercise physiological differences between children and adults which will not be reviewed here in detail. In short, during growth, children show a more marked increase in anaerobic metabolism than aerobic metabolism as they move through adolescence [18]. When children perform an increase in exercise work rate, the increase in VO_2_ to a new steady state (oxygen uptake kinetics) is much faster as in adults, and may be due to a more efficient oxygen delivery system, a greater relative capacity for oxygen utilization at the muscle, or both [18].

One of the more recently proposed parameters that can be obtained during exercise testing is the VE/VCO_2_ slope. The VE/VCO_2_ slope is obtained by linear regression analysis of ventilation (VE) to carbon dioxide exhalation (VCO_2_) as can be continuously measured during exercise during the complete exercise period. This value reflects ventilatory drive.

Another contemporary parameter that can be obtained during exercise testing is the Oxygen Uptake Efficiency Slope (OUES). In an attempt to develop an objective and effort-independent measure of cardiorespiratory fitness in children with CHD, the OUES was introduced [19]. The OUES represents the rate of increase of VO_2_ in response to a given VE during incremental exercise, indicating how effectively oxygen is extracted and taken into the body [20]. OUES is determined from the linear relation of VO_2_ (*y*-axis) versus the logarithm of VE (*x*-axis) during exercise, that is, VO_2_ = a log_10_ VE + b, where a is the OUES and b is the intercept [20]. The logarithmic transformation of VE is aimed at linearizing the otherwise curvilinear relation of VO_2_ versus VE, so making the OUES theoretically independent of the patient-achieved effort level. The OUES is a parameter that indicates the status of both systemic and pulmonary perfusion, and which explains the high correlation with VO_2peak_ [19, 21, 22].

## 4. Exercise Capacity in Patients with CHD

Currently, most patients with CHD will survive into adulthood. However, residual defects or problems occur relatively often. Although most patients will report normal exercise capacity, a reduction in exercise capacity may be the first sign of changes in cardiac function. Many cardiopulmonary variables may contribute to a reduced exercise capacity, including systolic and diastolic ventricular dysfunction, sinus node dysfunction, and changes in cardiac autonomic nervous activity. Many studies have investigated the exercise capacity by means of formal exercise testing in these patients [2, 23–31]. This is important, since many patients grossly overestimate their physical capabilities. There is only a poor association between the measured exercise capacity (e.g., peak oxygen uptake) and the self-reported physical functioning [6]. 

 Many studies investigating both children and adult patients with CHD found a lower than normal HR_peak_ [2, 23–31]. This lower HR_peak_ is usually defined as chronotropic incompetence when less than 80% of the predicted HR_peak_ is reached during graded exercise testing, although giving sufficient effort [23]. Factors influencing HR_peak_ in children are intrinsic sinus node dysfunction, and impaired sympathetic cardiac autonomic nervous activity [32] as well as the mode of exercise (e.g., running provides a somewhat higher HR_peak_ compared to cycling). HR dynamics seem to be more influenced by the surgeries itself than by the resultant hemodynamic abnormalities [33]. Cardiac denervation and damage to the sinus node or its blood supply, leading to sinus node dysfunction may play a role [33, 34]. Although parasympathetic nervous activity may improve after surgery, sympathetic reinnervation remains uncertain [35]. Chronotropic incompetence is also related to lower exercise capacity and increased levels of NT-pro-BNP in adults [36]. Disorders mostly related to chronotropic incompetence were single ventricle physiology (52%), Mustard operation for transposition of the great arteries (46%), and aortic coarctation (38%) [31], and Eisenmenger patients (90%) [23]. HR reserve is defined as the difference between resting HR and HR_peak_, and is physiologically important since, as described above, beyond moderate intensity exercise a further increase in CO can only be established by a further increase in HR. A decrease in HR reserve has been related to a higher mortality risk in adult CHD patients [23]. A slower HR recovery after cessation of exercise indicates impaired vagal autonomic activity and is usually related to a lower exercise capacity. This makes the HR recovery a good indication of vagal tone, and it has been shown to be a useful marker for evaluating patient outcomes in cardiac rehabilitation in children with CHD [2]. Cardiac autonomic nervous activity has also been shown to be useful in stratifying mild and severe heart failure in pediatric heart failure patients, due to dilated cardiomyopathy or as a result of CHD [33]. In some patients, for example, patients after Fontan operation, cardiac autonomic nervous activity has been found to be reduced even without signs of heart failure [37]. The role of the autonomic nervous system has been studied extensively by Ohuchi et al. [32, 38]. They found in patients with CHD a blunted HR increase during exercise, and delayed early HR recovery. These were independently associated with impaired sympathetic and parasympathetic cardiac autonomic nervous activity [38].

 When blood pressure is taken into account, maximal circulatory power can be defined as the peak work rate (Watt) × highest mean blood pressure that is reached during peak exercise. The use of circulatory power as a prognostic marker is independent from VO_2peak_ and chronotropic incompetence [24]. Peak circulatory power incorporates the blood pressure response to exercise, and it has been shown that the pressure-generating ability of the heart is a prognostic marker in heart failure patients [39]. In adult patients with CHD this circulatory power is reduced as well [24].

**Figure 1:**
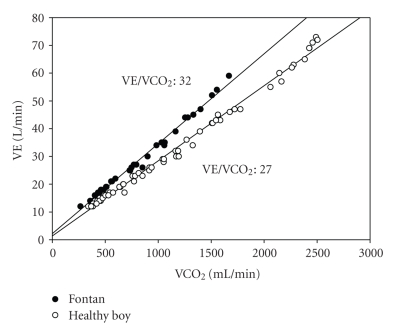
The VE/VCO_2_ slope in a 13 year old boy with a Fontan circulation and in a 13-year old healthy boy.

 The VO_2peak_ is reduced in most adult patients with CHD, ranging from a minor reduction in coarctation patients, to severely impaired VO_2peak_ in Eisenmenger patients [40]. The VE/VCO_2_ is increased in many CHD patient groups, being the worst in cyanotic patients with or without pulmonary hypertension [41]. However, some VSD patients may show normal values of VE/VCO_2_ [42]. The increase in VE/VCO_2_ slope indicates that in patients with CHD the ventilation is increased to constrain the fall in arterial pH. In Fontan patients an increased VE/VCO_2_ may be related to intracardiac and intrapulmonary shunting, and may be further impaired by increased pulmonary vascular resistance and low CO [41]. An example of a young Fontan patient is shown in Figure 1. In adults with cyanotic heart disease the VE/VCO_2_ slope was most closely related to symptoms of exercise incapacity [43].

 Pulmonary function may play an additional role in exercise limitations in some CHD patients [44]. Reductions in forced expiratory volume in 1 second, forced vital capacity, and 1-minute ventilatory volume have all been found in adults with CHD [45]. As lesions associated with increased pulmonary blood flow may result in pulmonary vascular obstructive disease, lesions with reduced pulmonary blood flow may result in hypoplasia of lung arteries and a reduced number of alveoli [45]. In operated patients thoracotomies and cardiopulmonary bypass can lead to increased lung stiffness and thereby further decrease pulmonary function [46].

**Figure 2:**
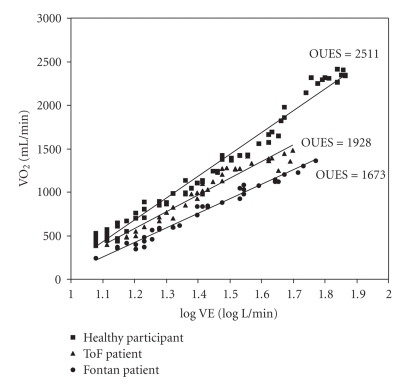
The OUES during an exercise test in a healthy 13-year-old boy, in a sex- and age-matched patient with tetralogy of Fallot, and in a sex- and age-matched Fontan patient.

 The oxygen uptake efficiency slope (OUES) has been used in adult heart failure patients, and has shown to be a reliable parameter, that can be obtained during submaximal exercise with a good correlation to VO_2peak_ [47]. Exercise training of these patients results in an improved OUES, also with a good correlation to the changes in VO_2peak_ [48]. However, OUES in adult cyanotic Fontan patients changes over the entire exercise duration, which makes it unable to predict maximal exercise capacity in these patients [49]. However, we observed a linear OUES values in pediatric Fontan patients with or without cyanosis as well as in pediatric tetralogy of Fallot patients (Bongers et al. submitted for publication). In addition we found significant differences between healthy children and children with CHD (with Fontan and tetralogy of Fallot repair). In Figure 2 an example of the OUES is provided.

## 5. Exercise Capacity in Specific Lesions

*Shunt Lesions*. In patients with a left to right shunt pulmonary blood flow (Qp) is increased in excess of systemic blood flow (Qs). A significant shunt is usually defined as a Qp/Qs ratio >2. The most common lesion with left to right shunting is an atrial septal defect (ASD), and there are significant differences in exercise capacity between patients before and after ASD closure. This contrasts to the unchanged exercise performance in VSD patients, although only small unoperated VSDs are included in the study of Binkhorst et al. [50]. They studied a small group of children who had undergone surgical closure of their VSD (*N* = 13) or had a small unoperated VSD (*N* = 14). Exercise capacity was not significantly different from controls. Although VSD patients had a lower participation in sports, and HR_peak_ was somewhat lower in the operated patients, these differences did not result in differences of peak work rate or VO_2peak_ as compared to healthy control subjects [50].

In a large group (*N* = 52) of adults (38.6 ± 15 years) with an ASD with a Qp/Qs of 2.7 ± 0.7, exercise capacity, was severely reduced before surgery [51]. Although surgery led to a significant improvement in exercise capacity as well as a decrease in VE/VCO_2_, in patients with preoperative signs of pulmonary hypertension exercise capacity remained below predicted values [51]. Also percutaneous ASD closure in adults led to an improvement in exercise capacity [52]. This increase was irrespective of age at ASD closure, but was absent in patients with a small atrial shunt (Qp/Qs<2). The improvement in VO_2peak_, and oxygen pulse (VO_2peak_/HR_peak_) was correlated with Qp/Qs before closure [53]. It was found that the improvement in VO_2peak_ and oxygen pulse were correlated to an increase in left ventricular ejection fraction and an increase in left ventricular enddiastolic diameter. It was concluded that an increase in both left ventricular SV and CO due to a positive ventricular interaction is the mechanism leading to improvement in VO_2peak_ [53]. In a study of Giardini et al. [54] 29 adults before, 6 and 36 months after transcatheter ASD closure were studied. A significant improvement in exercise capacity beyond 6 months post procedure was found. This was irrespective of age at intervention. The improvement was correlated to Qp/Qs before closure. The improvement was associated to an improvement in cardiac form and function. Those patients who did not reach normal values after 36 months (20%) had a severely reduced (<50% of predicted) VO_2peak_ before closure.

## 6. Pulmonary Valvar Stenosis

Patients who had surgical repair of an isolated pulmonary valvar stenosis during childhood show excellent long-term survival. Exercise capacity, however, is slightly decreased during long-term (22–33 years) followup as well as HR_peak_ [55]. This decrease is related to the development of pulmonary regurgitation [55]. Also long time after percutaneous balloon valvuloplasty of pulmonary stenosis the development of pulmonary regurgitation is associated with diminished exercise capacity and a lower VO_2peak_ [56]. In a group of children and adults with a variety of underlying CHD and pulmonary stenosis/regurgitation percutaneous pulmonary valve implantation led to an increase in exercise capacity, which was related to a reduction of pulmonary regurgitation [57]. From these studies it has become clear that especially the development of pulmonary regurgitation in former pulmonary stenosis patients has a deleterious effect on exercise capacity.

## 7. Fontan Circulation

Exercise capacity and cardiorespiratory responses to exercise are significantly reduced in patients who have undergone the Fontan procedure [58]. This appears to be due to the absence of a ventricle in the pulmonary circuit, disadvantageous systemic ventricular power, increased afterload profile, and a limited ventricular reserve capacity in these patients. Several studies have emphasized the ongoing risk for late failure and poor functional outcome. Ventricular filling, which is determined by the pulmonary vascular bed, appears to be a major determinant of the functional result after Fontan repair [59]. These findings have been extensively studied by Robbers-Visser et al. who determined that under dobutamine stress there was an abnormal decrease in end-diastolic volume, and no change in SV [60]. These findings further underscore the fact that in the Fontan patients there is an impaired preload during stress; therefore, CO (= SV × HR) can be increased only by increasing HR [60]. Interestingly, HR_peak_ during exercise is significantly reduced in Fontan patients. In a meta-analysis of 25 studies a mean HR_peak_ of 153 ± 10 beats/min was reported [61]. This low HR_peak_ further compromises increases in CO during exercise in Fontan patients.

 Recently, Muller et al. studied 57 patients after Fontan completion (age 8–52 years). Exercise capacity was severely reduced after total cavopulmonary connection (TCPC), corresponding to 60% of reference values [62]. This compares well with the results from 411 children with a Fontan circulation from 7 Pediatric Heart Network centers, who had a VO_2peak_ of 65% of predicted age and gender [63]. Fontan palliation in early childhood results in a higher VO_2peak_ during long-term followup [62]. Regular surveillance of the exercise capacity by spiroergometry is indispensable for the supervision of patients with Fontan haemodynamics [64]. In the study of Muller et al. daily activities of the Fontan patients was within recommended levels in 72% of the patients [62]. Daily activities were especially reduced in older patients and in patients with a lower VO_2peak_ [62]. Another study observed that the activity patterns in Fontan patients were markedly reduced as well. In a study of 147 Fontan patients, 7–18 years old, measured time spent in moderate and vigorous activity was markedly below normal at all ages, and was not significantly related to self-reported activity levels [4]. Lower physical activity levels were significantly related to lower perceived general health [4]. Since most patients with Fontan physiology have some degree of cyanosis, an increased hemoglobin concentration is necessary for adequate oxygen delivery to the tissues. As in other cyanotic lesions it is therefore essential to prevent iron deficiency which is directly related to exercise capacity [65].

## 8. Pulmonary Hypertension

CHD associated with large aortopulmonary shunt and high pulmonary pressure finally leads to irreversible pulmonary arterial hypertension. This situation carries a high risk of morbidity and mortality. Until recently treatment options were limited to the avoidance and treatment of complications. Patients with pulmonary arterial hypertension (PAH) related to CHD have an extremely low exercise capacity [66–68].

 The only options to improve exercise capacity is by reducing the pulmonary arterial hypertension. Bosentan treatment has been proven to induce short- and mid-term clinical, exercise, and haemodynamic improvements in patients with PAH related to CHD [66]. A small but significant improvement in VO_2peak_ was shown from 16.8 ± 1.4 to 18.3 ± 1.4 mL/kg/min [67]. However, objective exercise values appear to slowly return to baseline during longer follow-up periods [68]. The improvement in exercise capacity has been shown to be correlated to an improvement in quality of life and stabilization of exercise capacity.

## 9. Prognosis of Impaired Exercise Capacity

Although a mildly impaired exercise capacity may not interfere with normal daily life, its relation to prognostic values makes it an important monitoring tool. However, these prognostic markers have as yet only been found in adult CHD patients, while its role during childhood remains as yet speculative. A reduction in peak circulatory power is related to the presence of heart failure symptoms, and is a strong predictor of mortality [24]. Chronotropic incompetence is related to higher NYHA class, and increased NT-Pro-BNP [36], and predicts mortality independently of functional class and VO_2peak_ [23]. A reduction in VO_2peak_ predicts hospitalization and death [40]. Moreover, an abnormal VE/VCO_2_ slope is a strong predictor of death in adult patients with CHD [41].

## 10. Exercise Training in Congenital Heart Disease Patients

*Basic Exercise and Guidelines*. Children with CHD run the risk of becoming overweight and have low levels of physical activity [69]. A healthy and active lifestyle is as important in these patients as in the general population [70]. Regular physical activity is associated with many health benefits in patients with cardiac disease. Physical exercise and sports activities have an important beneficial effect on cardiorespiratory function. The intensity of exercise training should be adapted to the specific lesion and to the functional result obtained [71]. Advances in treatment have resulted in an increasing population of adults with CHD. Physical activity in these patients appears to convey beneficial effects, especially on health-related quality of life [3].

Many prepubertal children with CHD need no restrictions in their physical activity. Regular exercise at a recommended level can be performed and should be encouraged at all ages in all patients with CHD. Many children as well as adults can attend sports without any restriction [13, 72]. Special concern should be given to those patients with a significant ventricular dysfunction or recent history or risk of arrhythmia [72]. Although most patients show a willingness to participate in exercise, they frequently are uncertain about safety or benefit [73]. More than 50% of the patients show a significant lack of knowledge about appropriate levels of physical activity for their cardiac condition [74]. This may result in the fact that most adult patients fail to achieve national guidelines for physical activity, ranging from 77% in NYHA class I patients to 100% for those who are in NYHA class III and IV [73]. An important contributing factor to the impaired exercise capacity is the hypoactive lifestyle, as often observed in patients with CHD [75]. This frequently results from parental or environmental overprotection [76]. Patients with CHD should be actively encouraged and stimulated to adopt an active lifestyle including appropriate exercise training and sports [70]. General guidelines are provided in Table 3.

 In general, children with CHD should be advised to comply with public health recommendations of daily participation in 60 minutes or more of moderate to vigorous physical activity that is developmentally appropriate, enjoyable, and involves a variety of activities. Adults with CHD are advised to perform 30 minutes or more of moderate to vigorous activities on most days of the week.

## 11. How Effective Is Exercise Training?

Most studies in patients with CHD use an adult-type endurance exercise rehabilitation programme lasting ~3 months. Usually the training is performed 2-3 times per week, with an intensity between 60–80% of HR_peak_ [77]. After these training interventions an improvement in peak work rate [26, 27] and VO_2peak_ has been found [2, 26, 27]. This improvement persisted during a 6–12 months follow-up period [2, 27]. Also HR recovery during exercise improved [2]. The improvement in exercise capacity appeared to be a result of an increase in the oxygen pulse (=VO_2peak_/HR_peak_) at peak exercise, while significant changes in HR_peak_ were not observed [26, 27]. Exercise training may also improve respiratory muscle oxygenation in children with CHD [78]. These changes were associated with improvements in self-esteem, behavior, and emotional state [2]. Even a simple physical activity intervention like regular walking is feasible, safe, and significantly increases the exercise capacity of adult patients at all stages of CHD [5]. Also for Fontan patients it is possible to increase exercise capacity by a formal exercise training program [79]. In several studies with relatively small numbers of patients, VO_2peak_ and endurance time duration during exercise testing increased by 10–15%, while HR_peak_ remained unchanged [28, 79]. Even patients with severe chronic PAH can improve significantly in exercise capacity (6-minute walking distance, VAT and VO_2peak_) after a 15 week training program. In this program both moderate intensity (60–80% of HR_peak_) endurance exercise as well as low intensity resistance training and respiratory exercises as well were performed [80]. The results from this study show that formal exercise training is beneficial for patients with PAH. However, to our knowledge, no studies are available in pediatric patients with PAH related to CHD.

However, some words of caution are necessary, because there are many limitations in the designs of these studies. In many studies, there was no control group, a large drop-out of subjects and no long-term followup of the program. Future studies should overcome these methodologic shortcomings as well as design a more child-friendly exercise training program to enhance acceptability and enjoyment which will result in an improved adherence to the program and long-term exercise adherence.

## 12. Conclusion

In this paper, we discussed how exercise capacity can be measured by using only HR and work rate, or with the use of respiratory gas analysis in patients with CHD. In addition, we discussed the exercise capacity of patients with CHD, and possibilities of physical activity and exercise without considerable risks as well as exercise rehabilitation. Specific attention has been paid to a variety of CHD subgroups.

## Figures and Tables

**Table 1:** Indications for exercise testing in children.

	(1) Assesses physical capacity for recreational, athletic and occupational recommendations
	(2) Evaluates specific pathophysiologic characteristics
	(a) provides indications for surgery, therapy, or additional tests
	(b) evaluates functional postoperative success
	(c) diagnoses disease
	(3) Assesses adequacy of therapy
	(4) Assesses risk for future complications in existing disease
	(5) Instills confidence in child and parents
	(6) Motivates child for further exercise or weight loss

Modified after Bar-Or [11].

**Table 2:** Criteria for terminating exercise testing in children with CHD.

(1) Clinical	Symptoms as chest pain, severe headache, dizziness, chills, sustained nausea, inappropriate dyspnoea
	Signs as sustained pallor, clammy skin, disorientation, inappropriate affect
	Patient requests termination of the test

(2) Electrocardiography	Failure of heart rate to increase with exercise, and extreme fatigue, dizziness, or other symptoms suggestive of insufficient cardiac output
	Premature ventricular contractions (PVC) with increasing frequency
	Ventricular tachycardia (run of >3 PVCs)
	Supraventricular tachycardia
	ST segmental depression, or elevation, of more than 3 mm
	Triggering of atrioventricular (AV) block (2nd degree AV-block type Mobitz or 3rd degree AV block) by exercise
	Triggering of QTc lengthening >500 ms

(3) Blood pressure	Excessive levels (age dependent)—systolic blood pressure >250 mmHg, diastolic blood pressure >125 mmHg
	Progressive fall in systolic blood pressure with increasing work rate

(4) Progressive fall in oxygen saturation to <90% or a 10-point drop from resting saturation in a symptomatic patient	

Modified from Connuck [8] and Paridon et al. [12].

**Table 3:** Recommendations for competitive sport participation.

The following congenital heart defects can participate in all sports without restrictions
ASD (closed or small unoperated) and patent foramen ovale (Except Scuba diving in PFO)
VSD (closed or small unoperated)
AVSD (Only mild AV insufficiency; no significant subaortic stenosis or arrhythmia)
Partial or complete anomalous pulmonary venous connection (No significant pulmonary or systemic venous obstruction, no pulmonary
hypertension or exercise-induced arrhythmia)
Persistent ductus arteriosus (operated) (6 months post closure and no residual pulmonary hypertension)
Mild pulmonary stenosis (normal RV, normal ECG)
Mild aortic stenosis (With the exception of high static, high dynamic) (Mean gradient <21 mmHg; no history of arrhythmias, no dizziness,
syncope, or angina pectoris)
Transposition of the great arteries after arterial switch (With the exception of high static, high dynamic) (No or only mild neo-aortic
insufficiency; no significant pulmonary stenosis; no signs of ischemia or arrhythmia on exercise ECG)

Modified from Pelliccia et al. [13].

## References

[1] Deanfield J, Thaulow E, Warnes C (2003). Management of grown up congenital heart disease. The task force on the management of grown up congenital heart disease of the European Society of Cardiology. *European Heart Journal*.

[2] Singh TP, Curran TJ, Rhodes J (2007). Cardiac rehabilitation improves heart rate recovery following peak exercise in children with repaired congenital heart disease. *Pediatric Cardiology*.

[3] Giannakoulas G, Dimopoulos K (2009). Exercise training in congenital heart disease: should we follow the heart failure paradigm?. *International Journal of Cardiology*.

[4] McCrindle BW, Williams RV, Mital S (2007). Physical activity levels in children and adolescents are reduced after the Fontan procedure, independent of exercise capacity, and are associated with lower perceived general health. *Archives of Disease in Childhood*.

[5] Dua JS, Cooper AR, Fox KR, Stuart AG (2007). Physical activity levels in adults with congenital heart disease. *European Journal of Cardiovascular Prevention and Rehabilitation*.

[6] Gratz A, Hess J, Hager A (2009). Self-estimated physical functioning poorly predicts actual exercise capacity in adolescents and adults with congenital heart disease. *European Heart Journal*.

[7] Bergman AB, Stamm SJ (1967). The morbidity of cardiac nondisease in schoolchildren. *The New England Journal of Medicine*.

[8] Connuck DM (2005). The role of exercise stress testing in pediatric patients with heart disease. *Progress in Pediatric Cardiology*.

[9] Takken T, Blank AC, Hulzebos EH, Van Brussel M, Groen WG, Helders PJ (2009). Cardiopulmonary exercise testing in congenital heart disease: (contra)indications and interpretation. *Netherlands Heart Journal*.

[10] Takken T, Blank AC, Hulzebos EH, van Brussel M, Groen WG, Helders PJ (2009). Cardiopulmonary exercise testing in congenital heart disease: equipment and test protocols. *Netherlands Heart Journal*.

[11] Bar-Or O (1983). *Pediatric Sports Medicine for the Practitioner*.

[12] Paridon SM, Alpert BS, Boas SR (2006). Clinical stress testing in the pediatric age group: a statement from the American Heart Association council on cardiovascular disease in the young, committee on atherosclerosis, hypertension, and obesity in youth. *Circulation*.

[13] Pelliccia A, Fagard R, Bjørnstad HH (2005). Recommendations for competitive sports participation in athletes with cardiovascular disease: a consensus document from the Study Group of Sports Cardiology of the Working Group of Cardiac Rehabilitation and Exercise Physiology and the Working Group of Myocardial and Pericardial Diseases of the European Society of Cardiology. *European Heart Journal*.

[14] Singh TP, Rhodes J, Gauvreau K (2008). Determinants of heart rate recovery following exercise in children. *Medicine and Science in Sports and Exercise*.

[15] Tanaka H, Borres M, Thulesius O, Tamai H, Ericson MO, Lindblad L-E (2000). Blood pressure and cardiovascular autonomic function in healthy children and adolescents. *Journal of Pediatrics*.

[16] Albouaini K, Egred M, Alahmar A, Wright DJ (2007). Cardiopulmonary exercise testing and its application. *Heart*.

[17] ten Harkel ADJ, Takken T, Van Osch-Gevers M, Helbing WA Normal values for cardiopulmonary exercise testing in children.

[18] Armstrong N, Fawkner SG (2008). Non-invasive methods in paediatric exercise physiology. *Applied Physiology, Nutrition and Metabolism*.

[19] Baba R, Nagashima M, Goto M (1996). Oxygen uptake efficiency slope: a new index of cardiorespiratory functional reserve derived from the relation between oxygen uptake and minute ventilation during incremental exercise. *Journal of the American College of Cardiology*.

[20] Baba R, Nagashima M, Nagano Y, Ikoma M, Nishibata K (1999). Role of the oxygen uptake efficiency slope in evaluating exercise tolerance. *Archives of Disease in Childhood*.

[21] Marinov B, Mandadzhieva S, Kostianev S (2007). Oxygen-uptake efficiency slope in healthy 7- to 18-year-old children. *Pediatric Exercise Science*.

[22] Marinov B, Kostianev S (2003). Exercise performance and oxygen uptake efficiency slope in obese children performing standardized exercise. *Acta Physiologica et Pharmacologica Bulgarica*.

[23] Diller G-P, Dimopoulos K, Okonko D (2006). Heart rate response during exercise predicts survival in adults with congenital heart disease. *Journal of the American College of Cardiology*.

[24] Giardini A, Specchia S, Berton E (2007). Strong and independent prognostic value of peak circulatory power in adults with congenital heart disease. *American Heart Journal*.

[25] Fredriksen PM, Veldtman G, Hechter S (2001). Aerobic capacity in adults with various congenital heart diseases. *American Journal of Cardiology*.

[26] Rhodes J, Curran TJ, Camil L (2005). Impact of cardiac rehabilitation on the exercise function of children with serious congenital heart disease. *Pediatrics*.

[27] Rhodes J, Curran TJ, Camil L (2006). Sustained effects of cardiac rehabilitation in children with serious congenital heart disease. *Pediatrics*.

[28] Minamisawa S, Nakazawa M, Momma K, Imai Y, Satomi G (2001). Effect of aerobic training on exercise performance in patients after the Fontan operation. *American Journal of Cardiology*.

[29] Opocher F, Varnier M, Sanders SP (2005). Effects of aerobic exercise training in children after the Fontan operation. *American Journal of Cardiology*.

[30] Samman A, Schwerzmann M, Balint OH (2008). Exercise capacity and biventricular function in adult patients with repaired tetralogy of Fallot. *American Heart Journal*.

[31] Norozi K, Wessel A, Alpers V (2007). Chronotropic incompetence in adolescents and adults with congenital heart disease after cardiac surgery. *Journal of Cardiac Failure*.

[32] Ohuchi H, Watanabe K-I, Kishiki K, Wakisaka Y, Echigo S (2007). Heart rate dynamics during and after exercise in postoperative congenital heart disease patients. Their relation to cardiac autonomic nervous activity and intrinsic sinus node dysfunction. *American Heart Journal*.

[33] Ohuchi H, Takasugi H, Ohashi H (2003). Stratification of pediatric heart failure on the basis of neurohormonal and cardiac autonomic nervous activities in patients with congenital heart disease. *Circulation*.

[34] Ohuchi H, Tasato H, Sugiyama H, Arakaki Y, Kamiya T (1998). Responses of plasma norepinephrine and heart rate during exercise in patients after fontan operation and patients with residual right ventricular outflow tract obstruction after definitive reconstruction. *Pediatric Cardiology*.

[35] Ohuchi H, Suzuki H, Toyohara K (2000). Abnormal cardiac autonomic nervous activity after right ventricular outflow tract reconstruction. *Circulation*.

[36] Norozi K, Wessel A, Alpers V (2006). Incidence and risk distribution of heart failure in adolescents and adults with congenital heart disease after cardiac surgery. *American Journal of Cardiology*.

[37] Davos CH, Francis DP, Leenarts MFE (2003). Global impairment of cardiac autonomic nervous activity late after the Fontan operation. *Circulation*.

[38] Ohuchi H, Suzuki H, Yasuda K, Arakaki Y, Echigo S, Kamiya T (2000). Heart rate recovery after exercise and cardiac autonomic nervous activity in children. *Pediatric Research*.

[39] Ghali JK, Kadakia S, Bhatt A, Cooper R, Liao Y (1992). Survival of heart failure patients with preserved versus impaired systolic function: the prognostic implication of blood pressure. *American Heart Journal*.

[40] Diller G-P, Dimopoulos K, Okonko D (2005). Exercise intolerance in adult congenital heart disease: comparative severity, correlates, and prognostic implication. *Circulation*.

[41] Dimopoulos K, Okonko DO, Diller G-P (2006). Abnormal ventilatory response to exercise in adults with congenital heart disease relates to cyanosis and predicts survival. *Circulation*.

[42] Reybrouck T, Boshoff D, Vanhees L, Defoor J, Gewillig M (2004). Ventilatory response to exercise in patients after correction of cyanotic congenital heart disease: relation with clinical outcome after surgery. *Heart*.

[43] Gläser S, Opitz CF, Bauer U (2004). Assessment of symptoms and exercise capacity in cyanotic patients with congenital heart disease. *Chest*.

[44] Healy F, Hanna BD, Zinman R (2010). The impact of lung disease on the heart and cardiac disease on the lungs. *European Journal of Pediatrics*.

[45] Rigolin VH, Li JS, Hanson MW (1997). Role of right ventricular and pulmonary functional abnormalities in limiting exercise capacity in adults with congenital heart disease. *American Journal of Cardiology*.

[46] Habre W, Schütz N, Pellegrini M (2004). Preoperative pulmonary hemodynamics determines changes in airway and tissue mechanics following surgical repair of congenital heart diseases. *Pediatric Pulmonology*.

[47] Van Laethem C, Bartunek J, Goethals M, Nellens P, Andries E, Vanderheyden M (2005). Oxygen uptake efficiency slope, a new submaximal parameter in evaluating exercise capacity in chronic heart failure patients. *American Heart Journal*.

[48] van Laethem C, van de Veire N, De Backer G (2007). Response of the oxygen uptake efficiency slope to exercise training in patients with chronic heart failure. *European Journal of Heart Failure*.

[49] Giardini A, Specchia S, Gargiulo G, Sangiorgi D, Picchio FM (2009). Accuracy of oxygen uptake efficiency slope in adults with congenital heart disease. *International Journal of Cardiology*.

[50] Binkhorst M, van de Belt T, de Hoog M, van Dijk A, Schokking M, Hopman M (2008). Exercise capacity and participation of children with a ventricular septal defect. *American Journal of Cardiology*.

[51] Suchon E, Tracz W, Podolec P, Sadowski J (2005). Atrial septal defect in adults: echocardiography and cardiopulmonary exercise capacity associated with hemodynamics before and after surgical closure. *Interactive Cardiovascular and Thoracic Surgery*.

[52] Jategaonkar S, Scholtz W, Schmidt H, Fassbender D, Horstkotte D (2010). Cardiac remodeling and effects on exercise capacity after interventional closure of atrial septal defects in different adult age groups. *Clinical Research in Cardiology*.

[53] Giardini A, Donti A, Formigari R (2004). Determinants of cardiopulmonary functional improvement after transcatheter atrial septal defect closure in asymptomatic adults. *Journal of the American College of Cardiology*.

[54] Giardini A, Donti A, Specchia S, Formigari R, Oppido G, Picchio FM (2008). Long-term impact of transcatheter atrial septal defect closure in adults on cardiac function and exercise capacity. *International Journal of Cardiology*.

[55] Roos-Hesselink JW, Meijboom FJ, Spitaels SEC (2006). Long-term outcome after surgery for pulmonary stenosis (a longitudinal study of 22–33 years). *European Heart Journal*.

[56] Harrild DM, Powell AJ, Trang TX (2010). Long-term pulmonary regurgitation following balloon valvuloplasty for pulmonary stenosis. *Journal of the American College of Cardiology*.

[57] Khambadkone S, Coats L, Taylor A (2005). Percutaneous pulmonary valve implantation in humans: results in 59 consecutive patients. *Circulation*.

[58] Troutman WB, Barstow TJ, Galindo AJ, Cooper DM (1998). Abnormal dynamic cardiorespiratory responses to exercise in pediatric patients after fontan procudure. *Journal of the American College of Cardiology*.

[59] Gewillig MH, Lundstrom UR, Bull C, Wyse RKH, Deanfield JE (1990). Exercise responses in patients with congenital heart disease after Fontan repair: patterns and determinants of performance. *Journal of the American College of Cardiology*.

[60] Robbers-Visser D, ten Harkel DJ, Kapusta L (2008). Usefulness of cardiac magnetic resonance imaging combined with low-dose dobutamine stress to detect an abnormal ventricular stress response in children and young adults after Fontan operation at young age. *American Journal of Cardiology*.

[61] Takken T, Tacken MHP, Blank AC, Hulzebos EH, Strengers JLM, Helders PJM (2007). Exercise limitation in patients with Fontan circulation: a review. *Journal of Cardiovascular Medicine*.

[62] Müller J, Christov F, Schreiber C, Hess J, Hager A (2009). Exercise capacity, quality of life, and daily activity in the long-term follow-up of patients with univentricular heart and total cavopulmonary connection. *European Heart Journal*.

[63] Paridon SM, Mitchell PD, Colan SD (2008). A cross-sectional study of exercise performance during the first 2 decades of life after the Fontan operation. *Journal of the American College of Cardiology*.

[64] Ovroutski S, Ewert P, Miera O (2010). Long-term cardiopulmonary exercise capacity after modified Fontan operation. *European Journal of Cardio-thoracic Surgery*.

[65] Tay ELW, Peset A, Papaphylactou M Replacement therapy for iron deficiency improves exercise capacity and quality of life in patients with cyanotic congenital heart disease and/or the Eisenmenger syndrome.

[66] Duffels MGJ, Vis JC, van Loon RLE (2009). Effect of bosentan on exercise capacity and quality of life in adults with pulmonary arterial hypertension associated with congenital heart disease with and without Down’s syndrome. *American Journal of Cardiology*.

[67] Apostolopoulou SC, Manginas A, Cokkinos DV, Rammos S (2005). Effect of the oral endothelin antagonist bosentan on the clinical, exercise, and haemodynamic status of patients with pulmonary arterial hypertension related to congenital heart disease. *Heart*.

[68] Apostolopoulou SC, Manginas A, Cokkinos DV, Rammos S (2007). Long-term oral bosentan treatment in patients with pulmonary arterial hypertension related to congenital heart disease: a 2-year study. *Heart*.

[69] Pinto NM, Marino BS, Wernovsky G (2007). Obesity is a common comorbidity in children with congenital and acquired heart disease. *Pediatrics*.

[70] Thaulow E, Fredriksen PM (2004). Exercise and training in adults with congenital heart disease. *International Journal of Cardiology*.

[71] Picchio FM, Giardini A, Bonvicini M, Gargiulo G (2006). Can a child who has been operated on for congenital heart disease participate in sport and in which kind of sport?. *Journal of Cardiovascular Medicine*.

[72] Hirth A, Reybrouck T, Bjarnason-Wehrens B, Lawrenz W, Hoffmann A (2006). Recommendations for participation in competitive and leisure sports in patients with congenital heart disease: a consensus document. *European Journal of Cardiovascular Prevention and Rehabilitation*.

[73] Dua JS, Cooper AR, Fox KR, Stuart AG (2010). Exercise training in adults with congenital heart disease: feasibility and benefits. *International Journal of Cardiology*.

[74] Kendall L, Parsons JM, Sloper P, Lewin RJP (2007). A simple screening method for determining knowledge of the appropriate levels of activity and risk behaviour in young people with congenital cardiac conditions. *Cardiology in the Young*.

[75] Massin MM, Hövels-Gürich HH, Gérard P, Seghaye M-C (2006). Physical activity patterns of children after neonatal arterial switch operation. *Annals of Thoracic Surgery*.

[76] Reybrouck T, Mertens L (2005). Physical performance and physical activity in grown-up congenital heart disease. *European Journal of Cardiovascular Prevention and Rehabilitation*.

[77] Takken T, Hulzebos HJ, Takken T, van Brussel M, Hulzebos HJ (2008). Cardiac disorders. *Exercise Physiology in Children*.

[78] Moalla W, Maingourd Y, Gauthier R, Cahalin LP, Tabka Z, Ahmaidi S (2006). Effect of exercise training on respiratory muscle oxygenation in children with congenital heart disease. *European Journal of Cardiovascular Prevention and Rehabilitation*.

[79] Takken T, Hulzebos HJ, Blank AC, Tacken MHP, Helders PJM, Strengers JLM (2007). Exercise prescription for patients with a Fontan circulation: current evidence and future directions. *Netherlands Heart Journal*.

[80] Mereles D, Ehlken N, Kreuscher S (2006). Exercise and respiratory training improve exercise capacity and quality of life in patients with severe chronic pulmonary hypertension. *Circulation*.

